# Immunoglobulin G4-related disease of the thickened aortic valve extending to the left ventricular outflow tract causing severe aortic regurgitation and complete atrioventricular block: a case report

**DOI:** 10.1093/ehjcr/yty087

**Published:** 2018-07-31

**Authors:** Shumpei Kosugi, Masako Okada, Keiji Iwata, Shinji Hasegawa

**Affiliations:** 1 Department of Cardiology, Japan Community Healthcare Organization Osaka Hospital, 4-2-78 Fukushima, Fukushima-ku, Osaka, Japan; 2 Department of Clinical Laboratory, Japan Community Healthcare Organization Osaka Hospital, 4-2-78 Fukushima, Fukushima-ku, Osaka, Japan; 3 Department of Cardiovascular Surgery, Japan Community Healthcare Organization Osaka Hospital, 4-2-78 Fukushima, Fukushima-ku, Osaka, Japan

**Keywords:** IgG4-related disease, Aortic valve, Steroid therapy, Aortic regurgitation, Complete atrioventricular block, Heart failure, Case report

## Abstract

**Background:**

Immunoglobulin G4-related disease (IgG4-RD) is a systemic disease characterized by the tumefactive lesions and infiltration of IgG4-positive plasma cells. IgG4-RD has been described in various organs, but rarely the aortic valve. There are only a few reports of aortic stenosis, and none on significant aortic regurgitation. In addition, previous case reports relating to aortic valve lesions led to surgery as a first-line treatment. The effect of steroid treatment has not yet been determined.

**Case Summary:**

A 62-year-old man, receiving steroid therapy, who presented with general malaise, shortness of breath, and bradycardia. He had suspected IgG4-RD because of pancreatitis, lacrimal gland enlargement, and retroperitoneal fibrosis. An examination revealed a thickened aortic valve extending to the left ventricular outflow tract with severe aortic regurgitation and complete atrioventricular block. He received intensive steroid therapy for a suspected IgG4-related aortic valve lesion. The complete atrioventricular block improved, but worsening aortic regurgitation caused congestive heart failure. He required replacement of the aortic valve. A histopathological examination of the excised aortic valve leaflets revealed IgG4-positive lymphoplasmacytic infiltration with fibrotic tissue. The prosthetic valve was functioning well without leakage around the valve at the 1-year follow-up.

**Discussion:**

This case highlights the rare possibility that IgG4-RD of the aortic valve also causes significant aortic regurgitation. Conservative treatment with steroids may induce regression of the lesion and contribute to the stability of the prosthetic valve after surgery, but it may also exacerbate heart failure due to the progression of aortic regurgitation in patients with aortic valve lesions.

**Learning points**

- Immunoglobulin G4-related disease (IgG4-RD) of the aortic valve may cause aortic regurgitation and heart block. Steroid therapy is effective for regression of the lesion, but can exacerbate aortic regurgitation and cause heart failure.
- Regardless of whether serum IgG4 level normalizes, sustained increases in inflammatory markers during medication therapy for IgG4-RD may indicate the development of new lesions.

## Introduction

Immunoglobulin G4-related disease (IgG4-RD) is a recently recognized systemic disease characterized by the presence of numerous immunoglobulin G4 (IgG4)-positive plasma cells in lymphoplasmacytic infiltrates and fibrosclerotic tissue.^1^ IgG4-RD is described in almost every organ system, but rarely the aortic valve. There are only a few reports of IgG4-RD of the aortic valve, which was always associated with aortic stenosis.^2^^,^^3^

We report a patient with an IgG4-related aortic valve lesion that resulted in severe aortic regurgitation, a tumorous lesion in the left ventricular outflow tract (LVOT), and complete atrioventricular block. To our knowledge, this is the first report of a patient with IgG4-RD that developed mixed aortic valve disease with a prevalence of regurgitation and initially received conservative treatment with steroids before surgery.

## Timeline

**Table:** 

Day	Events
1	Patient presented with general malaise, shortness of breath, and bradycardia 1 year after the initiation of steroid (prednisolone 10 mg/day) administration under suspicion of immunoglobulin G4-related disease
	Thickened aortic valve with severe aortic regurgitation and complete atrioventricular block was revealed
8	Clinical diagnosis of IgG4-related aortic valve lesion
	High-dose steroid (methylprednisolone 1 g/day) therapy initiated for 3 days
11	Oral prednisolone dose increased to 30 mg/day
40	Oral prednisolone dose decreased to 25 mg/day
90	Administration of azathioprine (50 mg/day) initiated
100	Heart failure exacerbated due to worsening aortic regurgitation
125	Surgical aortic valve replacement performed
	Pathological diagnosis of IgG4-related aortic valve lesion

## Case presentation

A 62-year-old man with diabetes presented with general malaise, shortness of breath, and bradycardia. His medical history included retroperitoneal fibrosis, enlargement of the pancreas, and symmetrical swelling of the lacrimal glands with elevated serum IgG4 levels (175 mg/dL). He had received steroid therapy for 1 year for suspected IgG4-RD.

**Figure 1:**
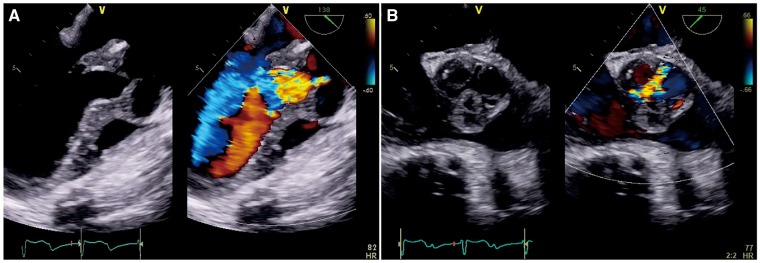
Transoesophageal echocardiography showing the thickened aortic valve with severe aortic regurgitation (*A*, aortic valve long-axis; *B*, aortic valve short-axis).

**Figure 2:**
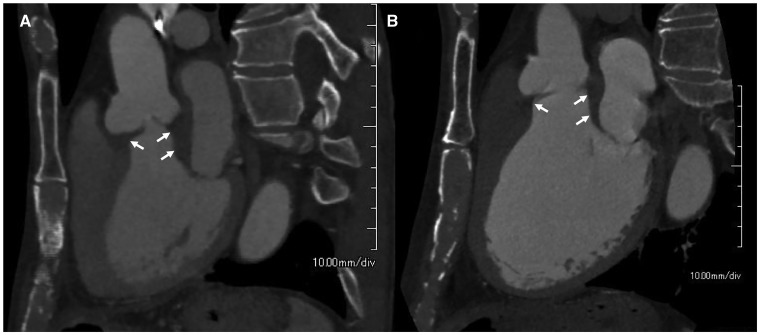
(*A*) Contrast-enhanced computed tomography at initial diagnosis showing the thickened aortic valve extending to the left ventricular outflow tract wall. (*B*) The preoperative contrast-enhanced computed tomography showing regression of the thickened aortic valve and left ventricular outflow tract wall.

A physical examination revealed a blood pressure of 90/42 mmHg, a pulse rate of 42 b.p.m., and body temperature of 36.6°C. An early diastolic murmur was noted on auscultation. The laboratory test results revealed elevated levels of white blood cells (WBC, 17 800/mm^3^), C-reactive protein (CRP, 4.07 mg/dL), and erythrocyte sedimentation rate (ESR, >120 mm) in the first hour. The serum IgG4 level was within the normal range (101 mg/dL) under oral prednisolone treatment (10 mg/day). An electrocardiogram revealed a complete atrioventricular block. A chest radiograph showed a normal cardiac silhouette and clear lung fields. Although echocardiography revealed a normal aortic valve 1 year ago, a transthoracic and transoesophageal echocardiography now revealed a thickened tricuspid aortic valve and the LVOT wall with severe aortic regurgitation, but no evidence of aortic stenosis and LVOT obstruction (*Figure 1*). The left ventricular (LV) systolic function was preserved, without wall motion abnormalities, and the LV end-diastolic diameter was slightly increased (*Table 1*). A contrast-enhanced computed tomography (CT) of the chest showed a thickened aortic valve extending to the LVOT wall and normal thickness of the ascending aortic wall (*Figure 2A*). A cardiovascular magnetic resonance (CMR) revealed a high-intensity signal around the aortic valve in the late gadolinium enhancement, whereas there was no significant change in the myocardium, the ascending aortic wall, and the surrounding structures.

**Table 1:** Time courses of echocardiographic parameters

Variables	Initial diagnosis	Preoperative
LVEF (%)	70	59
LVEDD (mm)	55	65
LVESD (mm)	33	44
LVOT diameter (mm)	23.1	23.9
AR jet width (%)	86	100
HDFR in descending aorta	Consistent	Consistent
AV peak velocity (m/s)	1.7	2.3
AV mean gradient (mmHg)	6	11

AR, aortic regurgitation; AV, aortic valve; LVEDD, left ventricular end-diastolic diameter; HDFR, holodiastolic flow reversal; LVEF, left ventricular ejection fraction; LVESD, left ventricular end-systolic diameter; LVOT, left ventricular outflow tract.

We increased the oral prednisolone to 30 mg/day after three days (1 g/day) of high-dose methylprednisolone for clinically suspected IgG4-RD of the aortic valve. The complete atrioventricular block improved to a first-degree atrioventricular block within a few days. Thus, he avoided permanent pacemaker implantation. He received an angiotensin receptor blocker (olmesartan 10 mg/day) and a long-acting loop diuretic (azosemide 60 mg/day) as a medical therapy for aortic regurgitation-related heart failure. The inflammatory markers CRP and ESR gradually returned to within the normal range, and his serum IgG4 level decreased further (56.3 mg/dL). A follow-up echocardiography demonstrated a slight regression of the thickened aortic valve and the LVOT wall. We thus decreased the oral prednisolone to 25 mg/day after 1 month of administration and initiated azathioprine (50 mg/day).^4^ A follow-up contrast-enhanced CT showed regression of the thickened aortic valve and the LVOT wall (*Figure 2B*).

Nevertheless, he presented with worsening shortness of breath and malaise three months after increasing the corticosteroid dose. Plasma brain natriuretic peptide increased from 69 pg/mL to 414 pg/mL, whereas the serum IgG4 level decreased to 27.6 mg/dL. The chest radiograph showed pulmonary congestion and cardiomegaly. The transthoracic echocardiography revealed the progression of aortic regurgitation and an enlarged LV dimension, whereas the aortic valve and LVOT wall thickening regressed. The LV systolic function was preserved, with no evidence of significant aortic stenosis and no LV wall motion abnormalities (*Table 1*).

**Figure 3:**
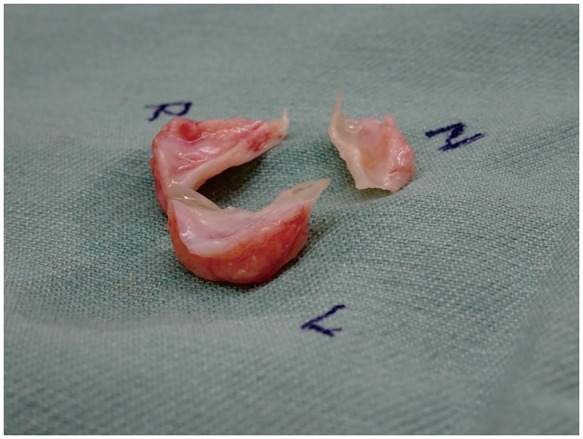
The excised aortic valve leaflets were shortened, with a maximum thickness of 8.0 mm.

**Figure 4:**
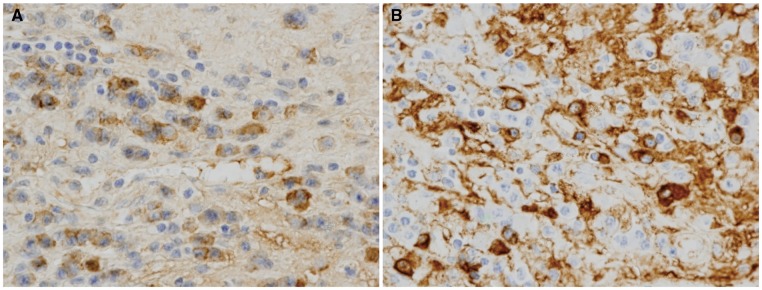
Immunostaining for (*A*) immunoglobulin G-positive plasma cells and (*B*) immunoglobulin G4-positive plasma cells (*A*, *B*: original magnification ×400).

He underwent aortic valve replacement for severe symptomatic aortic regurgitation and heart failure deterioration. The patient received a bioprosthetic aortic valve (25-mm Carpentier-Edwards PERIMOUNT; Edwards Lifesciences, Irvine, CA, USA), as we were concerned about the risk of anticoagulation-related bleeding and the possibility that there could be problems further down the line with complications from multiple organs and ongoing anticoagulation. During the operation, we observed the thickening and shortening of the tricuspid aortic valve (*Figure 3*). The entire LVOT wall was also thickened. In contrast, the ascending aorta was normal. The pathological examination of the excised valve leaflets showed a dense lymphoplasmacytic infiltrate mixed with fibrotic tissue. The immunohistochemical staining revealed a ratio of IgG4-positive plasma cells to IgG-positive plasma cells greater than 0.5 (*Figure 4*). Thus, he was diagnosed with IgG4-RD of the aortic valve.

The dose of prednisolone was tapered by 2.5 mg every 2 months in combination with azathioprine 50 mg after surgery. The transthoracic echocardiography 1 year after the surgery showed regression of the LV dilatation and normal function of the prosthetic valve.

## Discussion

IgG4-RD is a newly recognized systemic disease characterized by tumefactive lesions containing lymphoplasmacytic infiltrate and sclerosis, rich in IgG4-positive plasma cells.^1^ IgG4-RD was initially reported in 2001 in sclerosing pancreatitis associated with increased levels of serum IgG4.^5^ Since this original description, IgG4-RD has been described in various organs such as the biliary tree, salivary glands, kidneys, lungs, aorta, and others.^6^ Cardiovascular involvement may manifest as aortitis, inflammatory aneurysm, periaortitis, cardiac pseudotumors, coronary arteritis and pericarditis, but the aortic valve is rarely affected.^7^ Although previous reports have shown that aortic valve lesions are associated with aortic stenosis, our patient developed only aortic regurgitation and not significant aortic stenosis.^2^^,^^3^ Furthermore, in previously reported cases, surgical procedures, mainly aortic valve replacement, were performed first. To the best of our knowledge, this is the first report of a case treated with steroid therapy before surgery. Yamauchi *et al.*^8^ presented a patient with a pseudotumour in the left atrium, causing aortic regurgitation and heart block, who also underwent aortic valve replacement before steroid administration.

One of the potential problems related to valve surgery without initial steroid therapy in these individuals is the possibility of leakage and early prosthetic valve failure. Maleszewski *et al.*^2^ reported a case, in which aortic valve replacement was performed for aortic stenosis and regurgitation without preoperative steroid therapy, that developed perivalvular leakage approximately 1 year after surgery. Yamauchi *et al.*^8^ presented a case in which steroid treatment was started after aortic valve replacement that required redo surgery because of the perivalvular leakage 8 months after the operation. They considered that perivalvular leakage might have resulted from the loosening of the sutures placed in the annulus due to regression of the tumefactive lesion after the initiation of steroid therapy. In our patient, the prosthetic valve was functioning well without leakage around the valve at the 1-year follow-up. The preoperative steroid administration may have contributed to the stability of the prosthetic valve.

Steroid therapy is widely recognized as the first-line therapy for IgG4-RD. A consensus statement from Japan suggested treating patients initially with prednisolone at a dose of 0.6 mg/kg of body weight.^4^ According to the literature, our patient was started on steroid treatment when he was suspected to have IgG4-related pancreatitis, lacrimal gland enlargement, and retroperitoneal fibrosis approximately 1 year ago. The prednisolone was tapered to 10 mg/day without recurrence of the already known lesions. Although his serum IgG4 level gradually decreased to within the normal range, inflammatory markers (CRP, ESR, and WBC) remained elevated. We should have assessed not only the serum IgG4 level but also inflammatory markers while tapering the steroid doses, to detect the development of new lesions. Our patient responded well to increased prednisolone. The thickening of the aortic valve leaflets regressed due to the intensive steroid therapy after the development of the aortic valve lesion. Heart failure was exacerbated, however, by the progression of aortic regurgitation. The suspected cause of aortic regurgitation, based on echocardiographic findings, is that the shortening and thickening of the valve leaflets might have caused a coaptation failure (*Figure 1B*). We theorise that changes in the geometry of the aortic valve, resulting from a reduction in the inflammatory infiltrate by steroid therapy, may have exacerbated the aortic regurgitation due to the coaptation failure and, subsequently, the LV dilatation.

Multimodality imaging, including echocardiography, multidetector CT, and CMR, has been used for early diagnosis and follow-up of IgG4-related cardiovascular disease.^7^^,^^9^ An echocardiography is particularly useful for the evaluation of valvular involvement as in our patient. It provides structural and functional information about the aortic valve and enables early diagnosis and severity assessment of valvular disease, and is also essential to evaluate cardiac function and heart failure. A multidetector CT allows multiplanar reformation; thus, allowing evaluation of the aortic valve, aortic wall, and myocardium changes. Although the spatial resolution of a CMR is inferior to a CT, a CMR has superior soft tissue characterization, and can evaluate disease activity and response to treatment due to the ability to characterize tissue oedema and fibrosis.

In conclusion, we describe a case of steroid therapy preceding surgery for IgG4-RD of the aortic valve that caused severe aortic regurgitation and complete atrioventricular block. Preoperative steroid treatment may induce regression of the lesion and contribute to the stability of the prosthetic valve after surgery, but it may also carry a risk of heart failure exacerbation due to the progression of aortic regurgitation in patients with aortic valve lesions.

**Consent:** The author/s confirm that written consent for submission and publication of this case report including image(s) and associated text has been obtained from the patient in line with COPE guidance.

**Conflict of interest:** none declared.
